# Characterizing reproducibility of cerebral hemodynamic responses when applying short-channel regression in functional near-infrared spectroscopy

**DOI:** 10.1117/1.NPh.9.1.015004

**Published:** 2022-03-07

**Authors:** Dominik G. Wyser, Christoph M. Kanzler, Lena Salzmann, Olivier Lambercy, Martin Wolf, Felix Scholkmann, Roger Gassert

**Affiliations:** a ETH Zurich, Rehabilitation Engineering Laboratory, Department of Health Sciences and Technology, Zurich, Switzerland; b University Hospital Zurich, University of Zurich, Biomedical Optics Research Laboratory, Department of Neonatology, Zurich, Switzerland; c University of Bern, Institute of Complementary and Integrative Medicine, Bern, Switzerland

**Keywords:** functional near-infrared spectroscopy, reproducibility, short-channel regression, intraclass correlation, brain–computer interface

## Abstract

**Significance:**

Functional near-infrared spectroscopy (fNIRS) enables the measurement of brain activity noninvasively. Optical neuroimaging with fNIRS has been shown to be reproducible on the group level and hence is an excellent research tool, but the reproducibility on the single-subject level is still insufficient, challenging the use for clinical applications.

**Aim:**

We investigated the effect of short-channel regression (SCR) as an approach to obtain fNIRS measurements with higher reproducibility on a single-subject level. SCR simultaneously considers contributions from long- and short-separation channels and removes confounding physiological changes through the regression of the short-separation channel information.

**Approach:**

We performed a test-retest study with a hand grasping task in 15 healthy subjects using a wearable fNIRS device, optoHIVE. Relevant brain regions were localized with transcranial magnetic stimulation to ensure correct placement of the optodes. Reproducibility was assessed by intraclass correlation, correlation analysis, mixed effects modeling, and classification accuracy of the hand grasping task. Further, we characterized the influence of SCR on reproducibility.

**Results:**

We found a high reproducibility of fNIRS measurements on a single-subject level (${\mathrm{ICC}}_{\text{single}}=0.81$ and correlation r=0.81). SCR increased the reproducibility from 0.64 to 0.81 (${\mathrm{ICC}}_{\text{single}}$) but did not affect classification (85% overall accuracy). Significant intersubject variability in the reproducibility was observed and was explained by Mayer wave oscillations and low raw signal strength. The raw signal-to-noise ratio (threshold at 40 dB) allowed for distinguishing between persons with weak and strong activations.

**Conclusions:**

We report, for the first time, that fNIRS measurements are reproducible on a single-subject level using our optoHIVE fNIRS system and that SCR improves reproducibility. In addition, we give a benchmark to easily assess the ability of a subject to elicit sufficiently strong hemodynamic responses. With these insights, we pave the way for the reliable use of fNIRS neuroimaging in single subjects for neuroscientific research and clinical applications.

## 1 Introduction

As an optical and noninvasive technology to capture concentration changes of oxyhemoglobin ($O_{2}\mathrm{Hb}$) and deoxyhemoglobin (HHb), functional near-infrared spectroscopy (fNIRS) is an established technique for measuring cerebral hemodynamic changes associated with brain activity.^1^^–^^4^ It enables the measurement of changes in cerebral hemodynamics that are associated with task-related brain activity patterns.^5^ In recent years, the application of fNIRS started transitioning from controlled research laboratories to more natural environments and real-world tasks.^6^^–^^8^ The wearable and unconstrained use of fNIRS paves the way for neuroimaging applications, for example, for bedside and in-home monitoring of brain function^9^^,^^10^ or for brain–computer interface (BCI) settings to assist neurologically impaired persons during activities of daily living when combined with robotic devices.^11^^–^^13^ Such applications call for cutting-edge fNIRS systems that fulfill high requirements regarding technology [e.g., high signal-to-noise ratio (SNR), fast signal processing, and features to remove movement artifacts] and usability (e.g., high comfort and accurate sensor placement) to capture small changes in brain activity in daily life settings.^7^^,^^14^ Further, the in-home and clinical monitoring of brain activity places strong requirements on the robustness and reliability/reproducibility of fNIRS measurements as these factors directly affect the ability to sensitively capture neurological changes and to accurately control external devices with a BCI. Although good reproducibility has been found on group level,^15^^–^^17^ which is sufficient to answer many research questions, the proof of reproducible fNIRS measurements across multiple days for individuals has not been given. Because the single-level reproducibility is of fundamental importance for most clinical and everyday applications, thoroughly characterizing it is essential.

The main factors that are expected to affect the reproducibility of fNIRS measurements are the signal quality of the hardware (i.e., SNR),^18^^,^^19^ the placement and fixation of the optodes,^18^^,^^20^ and the presence and variability of physiological changes.^16^^,^^21^^,^^22^ Although the first two points are expected to be addressable through cutting-edge fNIRS hardware, for example, using photodetectors^23^ and advanced source localization and optode placement techniques, such as transcranial magnetic stimulation (TMS) guided fNIRS,^24^ addressing physiological changes remains a major challenge. More specifically, the interfering physiological influences in fNIRS are a multifaceted combination of different physiological signals. For example, Mayer waves (MWs) or task-evoked hemodynamic changes due to the sympathetic activation of the autonomic nervous system are present in different tissue layers that are penetrated by the near-infrared light (i.e., scalp and brain).^25^^–^^27^ To attenuate the confounding effect of physiological changes in fNIRS measurements, advanced signal processing techniques, such as short-channel regression (SCR),^28^ are required. With the SCR approach, a regressor signal obtained from a short channel (ideally <8  mm^29^) measurement is subtracted from a long channel (∼30  mm for adults) measurement. The short channel predominantly contains extracerebral (i.e., scalp) information and enables the removal of physiological changes from the long-channel measurement, which is a combination of cerebral and extracerebral signals.^28^^,^^30^^,^^31^ However, systematic investigations into the effect of SCR on the reproducibility of fNIRS measurements are lacking.

The aim of this paper is to provide an fNIRS measurement approach that allows for capturing cerebral hemodynamic responses with high reproducibility on an individual level. Furthermore, we quantify the effect of SCR on the hemodynamic response and its link to measurement reproducibility, brain activity estimates, and BCI classification using fNIRS. This work is important as it addresses the fundamental challenge of single-subject reproducibility, which is a crucial point for establishing fNIRS as a neuroimaging technique, and helps to translate fNIRS into daily life environments.

## 2 Materials and Methods

### 2.1 Participants

Fifteen subjects (9 males and 6 females, Caucasian, mean age±SD: 27±4.6  years) participated in our study. Only subjects that fulfilled all inclusion criteria for the use of TMS^32^ were recruited. Subjects 2, 4, and 15 were left-handed, the other twelve subjects were right-handed. The body-mass index was calculated for every subject, and hair root density, hair color. and hair thickness were assessed on a scale from 0 to 5 (0: no hairs, 5: dense, thick, dark hairs) upon visual inspection by the same experimenter (LS).

The experiments were approved by the ethical commission of ETH Zurich (2018-N-22), the Cantonal Ethics Commission of the Canton of Zurich (2018-01078), and were conducted in accordance with the Declaration of Helsinki. Written informed content was provided by all subjects.

### 2.2 fNIRS Instrumentation

**Fig. 1:**
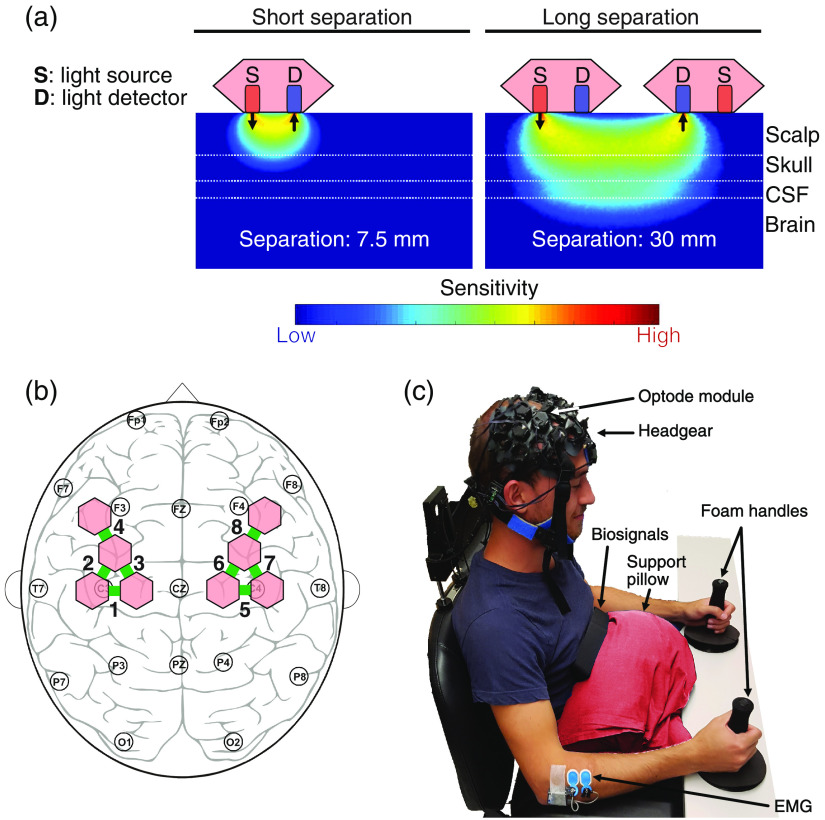
Experimental setup. (a) Sensitivity maps of SS and long-separation measurements for two optodes. Two-dimensional sensitivity maps were obtained from Monte Carlo simulations using ValoMC.^29^^,^^34^^,^^35^ (b) Arrangement of the optoHIVE optodes (red) and ROIs (green) according to the 10–20 system of EEG placement. Each ROI consists of two long-separation channels that probed the same brain regions. (c) Subjects were seated with arms resting comfortably on cushioned armrests and the hands grasping a foam handlebar. If desired, a support pillow was added to additionally support the arms. OptoHIVE was placed over the left and right motor areas to record fNIRS signals. Different biosignals were concomitantly acquired (not used in this work).

A custom-built fNIRS instrument called optoHIVE was used to detect cortical brain activity.^23^^,^^33^ It is a lightweight, fiber-less system designed for wearable, high-quality measurements, and each optode includes a four-wavelength LED light source (774, 817, 865, and 892 nm) and a silicon photomultiplier for photodetection.^33^ This system was preferred over commercial fNIRS instruments because, in addition to wearability, it offers the advantages of modular optode placement, a large number of short-distance channels, and high optical sensitivity.^23^ Each optode module (containing a light source and detector) comprises a short-separation (SS) channel of 7.5 mm and measures with every other optode within 30 mm distance, as shown in Fig. 1(a). In this study, eight optode modules were fixed using a custom-built headgear made of silicone patches, three-dimensional-printed parts, and elastic strings [see Fig. 1(c)]. The optodes were symmetrically placed over the left and right primary motor cortices (M1), ventral premotor cortices, and dorsal premotor cortices, resulting in 16 long-separation (LS) channels of 30 mm and 8 SS (7.5 mm) channels. As always, two channels measured from the same brain location (i.e., their light-paths were overlapping), and the 16 channels were reduced to eight ROIs. A map of the optode configuration and the ROIs is shown in Fig. 1(b). Measurements performed by optoHIVE are controlled through a LabVIEW (Version 2015, National Instruments NI, Texas) interface, and data from each channel are collected at a sampling frequency of 8.98 Hz over an NI myRIO data acquisition device.^33^

### 2.3 Study Protocol

#### 2.3.1 M1 localization with TMS

To minimize the influence of variations in the optode placement at different measurement days and to maximize the sensitivity to the targeted M1 brain region, we determined the locations of the left and right M1 via TMS at the beginning of each session and marked it with a dry marker. Single-pulsed TMS was performed with a 70-mm Magstim200 stimulator (Magstim Company Ltd., Wales, United Kingdom) and a figure-eight TMS coil. The coil was placed tangentially to the scalp with the handle in the dorsal direction and laterally at 45 deg away from the mid-sagittal line. During the magnetic stimulation, electromyography (EMG) activity (Bagnoli, Delsys, CH) of the contralateral abductor pollicis brevis muscle was monitored.^36^ The location with the strongest motor-evoked-potential response was determined as the “hotspot” for the hand grasping representation.

#### 2.3.2 fNIRS study protocol

All 15 subjects completed a test-retest protocol consisting of two sessions on two different days [time-span mean±SD (min–max): 5.5±3.1 (1 to 13) days]. Subjects were seated comfortably in front of a computer screen, with their elbows resting on cushioned armrests (if desired, pillows were added for comfort). The left and right hands were placed in an upright position around a custom-built handlebar surrounded with foam [see Fig. 1(c)]. The task was defined as a self-paced, active left hand or right hand grasping task (isometric grasping) at a frequency of ∼1  Hz. The grasping task was trained with a visual display and an auditory metronome at the beginning of each session. Each of the two sessions included two runs in which the subject repeatedly performed either the left or the right hand grasping task. An arrow on the screen pointing left or right indicated the hand that had to be moved (i.e., left or right hand grasping). Each run included a block design protocol with 30 trials (15 left and 15 right) of 16 s and a randomized interstimulus duration between 15 and 24 s. A text display announced the upcoming task 2 s before each trial. At the beginning and end of each run, a baseline of 120 and 60 s, respectively, was added with the subject remaining at rest. Subjects were instructed to refrain from any movement during the run other than the instructed grasping movements.

### 2.4 Data Processing

Data processing was performed in MATLAB (R2017a, Mathworks Inc.). Motion artifacts were removed using spline interpolation.^37^ Raw optical intensities were converted to concentration changes of $O_{2}\mathrm{Hb}$ ($\left[O_{2}\mathrm{Hb}\right]$) and HHb ([HHb]) using the modified Beer–Lambert law. The absorption coefficients were adopted from Moaveni,^38^^,^^39^ and the differential pathlength factors for the four wavelengths (6.2, 6.2, 5.9, 5.5) were from Cope.^40^ For the removal of drift and cardiac pulsation of $\left[O_{2}\mathrm{Hb}\right]$ and [HHb], different methods were considered according to Pinti et al.,^41^ but the best results were achieved following their suggested optimal filter (finite impulse response, order 1000) at cutoff frequencies of 0.015 and 0.35 Hz. Building on our previous work,^27^ multichannel SCR based on non-negative least squares (${\mathrm{GLM}}^{\mathrm{multiSS}}$) was applied to best reduce the influence of physiological changes and, thus, to separate the hemodynamic changes from the extracerebral tissue layer. With ${\mathrm{GLM}}^{\mathrm{multiSS}}$, all short-channel distances are included as regressors in the general linear model (GLM) with the precautional measure of allowing for only positive estimates (non-negative least squares regression). Short channel signal quality was verified following the approach of Perdue et al.,^42^ which is based on the signal content (heart rate) rather than purely on the SNR.^23^ It was found that 90% of the short channels were of good quality during our measurements when considering a signal quality threshold of 12 dB.^27^ Amplitudes of MW oscillations were obtained from [$O_{2}\mathrm{Hb}$] by normalizing the band-power (0.07 to 0.14 Hz) with its pulse band-power (0.6 to 2 Hz), and the median value of all long-separation channels was extracted.^27^

A GLM^27^^,^^43^ was applied on the time course of the long-separation channel (i.e., with and without SCR). As an evaluation metric, t-values were obtained. The t-values give an indication on the signal strength of a fitted hemodynamic response curve in relation to the residuals. The used GLM consisted of a modeled hemodynamic response time course, obtained from the convolution of the boxcar function and the canonical hemodynamic response,^43^ its time and dispersion derivatives, and a constant offset, which were fitted into the fNIRS data of each recording channel. The time and dispersion derivatives^44^ were included to correct for deviations of the onset and the shape of the hemodynamic response, respectively. The t-values were stored in a vector with 1920 entries (15 subjects × 16 channels × 2 hands × 2 runs × 2 sessions) for [$O_{2}\mathrm{Hb}$] and [HHb]. When ROI analysis was performed, the average of the t-values of the corresponding channels was used. GLM analysis was applied on non-regressed (NR) long-separation measurements and on SCR data.

### 2.5 Statistical Analysis

Statistical analysis was performed in R (Version 3.6.3, RStudio Inc.).^45^ To find a threshold to distinguish between active and inactive channels, GLM was applied on the baseline data (i.e., random task onsets during rest condition without systemic brain activity), and t-values were extracted. From the obtained t-values distributed around 0, the threshold, below which the probability is >95% that the brain is in rest condition, was extracted. Consequently, the 5% significance level to indicate if a hemodynamic response, representing brain activity above chance, was present, was found to be t≥30 for SCR data and t≥22 for unregressed data.

Reproducibility between sessions was assessed using linear correlation analysis and intraclass correlation coefficient (ICC) analysis applied to the t-values provided by the GLM. Linear correlation between sessions 1 and 2 on a group level was calculated based on Pearson correlation coefficients applied on the t-values of the M1. For this purpose, the t-values for left M1 (right hand task) and right M1 (left hand task) were extracted, averaged for the two runs per session, and correlated between sessions for the 15 subjects. Test-retest reliability was determined using ICC based on an absolute agreement, two-way random effects model with repeated measures.^17^^,^^46^ Single (ICC(2,1)) and average (ICC(2,*k*)) measures and their 95% confidence intervals were calculated as suggested by Li et al.^17^ The ICC gives an indication of the reliability of measurements by comparing the variability of different tests of the same individuals with the total variation across all ratings and all individuals. A high ICC (close to 1) indicates low intrasubject variability relative to the intersubject variability, whereas a low ICC (close to 0) means that values from the same group are not similar.^46^ Thresholds for interpreting ICCs vary in literature; we used the definition according to Li et al.:^17^ poor (ICC<0.40), fair (0.40≤ICC<0.60), good (0.60≤0.75), and excellent (0.75≤ICC<1.00). To estimate the change in t-values across measurement sessions, the mean absolute scaled error (MAE%) between sessions 2 and 1 was calculated and normalized with respect to the range of observed values.

A linear mixed effects model with restricted maximum likelihood estimation (*lmer* in R) was applied on the t-values to investigate the statistical significance of factors that could affect the estimation of brain activation. Two mixed effects models were established, one for [$O_{2}\mathrm{Hb}$] and one for [HHb]. The dependent variable consisted of the 1920 t-values (channelwise). The fixed effects were selected as (1) the interaction between hand and channel hand*channel, (2) run, (3) raw signal strength, and (4) MW amplitude. As random variables, an intercept for subject and a nested random effect of channel per subject were considered to allow levels of t-value to vary across channels and subjects. Square-root transformation was applied to ensure that the model residuals were normally distributed. The goodness of fit was verified from a normal distribution and homoscedasticity of the model residuals. After model fitting, the estimates were backtransformed, and their effect was investigated by multiple comparisons with Tukey contrasts.^47^^,^^48^

### 2.6 Classification

A classifier was trained and tested on the obtained fNIRS data to link the aspect of reproducibility with a potential application such as a BCI. Therefore, pseudo-online classification (i.e., continuous samplewise processing of data) was performed using a support vector machine (SVM) with L1-norm regularization^49^ to classify between right and left hand grasping. First, [$O_{2}\mathrm{Hb}$] and [HHb] were bandpass-filtered with a forward filter between the cutoff frequencies 0.005 Hz (Chebyshev type II, order 2) and 0.35 Hz (Butterworth, order 4). Second, adaptive filtering based on non-negative weight estimation was used for the samplewise regression (i.e., SCR) of the SS channels from the long-separation channels. Third, feature extraction was performed by extracting three feature types from [$O_{2}\mathrm{Hb}$] and [HHb]: amplitude, slope, and correlation-based signal improvement.^50^ Fourth, an L1-norm SVM was used for joint training of the classifier and feature selection.^51^ Its regularization parameter was found with fivefold cross-validation on the training data. The classifier was trained on the first run and tested on the second run of the same session. The intrasession accuracy was calculated over the 30 trials per testing run for sessions 1 and 2, separately. The entire 16 s trial window was used for classification, and a decision was obtained at the end of each trial. Although other window sizes or features could be considered, we selected the entire duration to keep the processing pipeline simple as similar results would be expected with a shorter window and delay until a decision is made was not deemed critical during this study. Three classifier scenarios were trained to investigate the effect of the selected dataset (i.e., SS signals only, long-separation only, or SCR signals) on classification accuracy.

## 3 Results

### 3.1 Spatial Activation Patterns

**Fig. 2:**
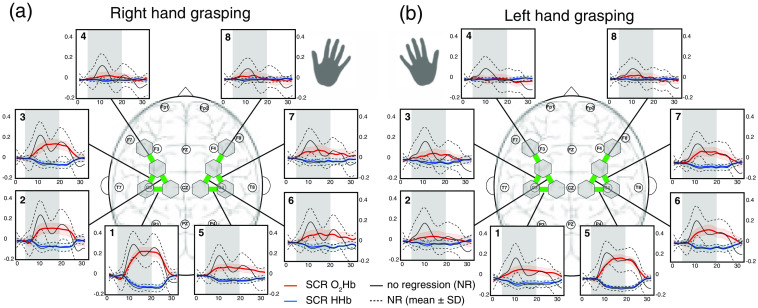
Group average of [$O_{2}\mathrm{Hb}$] and [HHb]. The hemodynamic responses of the 15 subjects were averaged (mean±SD) for each ROI for (a) the right and (b) left hand grasping tasks. The gray bars indicate the task period when the grasping task was conducted. The colored lines represent relative concentration changes of $O_{2}\mathrm{Hb}$ (red) and HHb (blue) when SCR was applied, with the shaded areas indicating the standard deviation (SD). The black (dotted) lines represent the mean (and SD) of the unregressed (NR) signal. The numbers in the upper left corners indicate the ROI. Units are in μM.

On a group level, spatially specific patterns of brain activity were detected for the left and right grasping tasks, shown in Fig. 2. The strongest hemodynamic response for the right hand task was observed over the contralateral (left) M1 and vice versa for the left hand task. In particular, ROI 1 showed the strongest magnitude for the right hand task with an average change of 0.26/−0.12  μM for [$O_{2}\mathrm{Hb}$] and [HHb], whereas for the left hand task, ROI 5 changed the strongest with 0.21/−0.09  μM ([$\left[O_{2}\mathrm{Hb}]/[\mathrm{HHb}\right]$). Weaker activation was observed at the adjacent frontal brain areas (ROI 2+3 for the right hand and ROI 6+7 for the left hand) and the ipsilateral M1 (ROI 5 for the right hand and ROI 1 for the left hand). The remaining ROI (ROI 4+6+7+8 for the right hand task and ROI 2+3+4+8 for the left hand task) did not exhibit significant activation. The hemodynamic responses for the NR signals had slightly larger magnitudes mainly for [$O_{2}\mathrm{Hb}$] than for the SCR signals with 0.33/−0.13  μM versus 0.26/0.12  μM for the right hand task and 0.27/−0.10μM versus 0.26/0.09  μM for the left hand task. This observation was accompanied by a smaller standard deviation of the signal. In comparison with the NR signal, the SCR signal did not exhibit an oscillatory response after task onset.

**Fig. 3:**
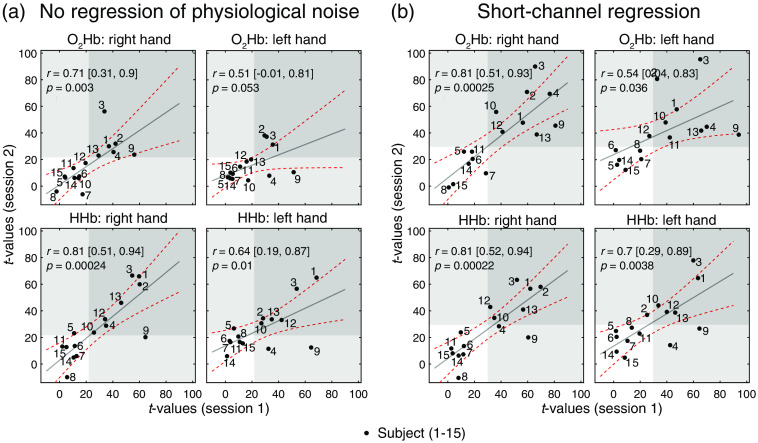
Correlation between test and retest sessions. Reproducibility of t-values between the two sessions was assessed for [$O_{2}\mathrm{Hb}$] and [HHb] of the right and left hand grasping tasks. t-values give an indication of the quality of the measured hemodynamic response. For each task, the correlation plots for the ROIs over the contralateral M1 (i.e., left M1 for right hand grasping and right M1 for left hand grasping) are shown. (a) Results for NR t-values. (b) Results for regressed (after SCR). Bright and dark gray areas indicate that the t-values exceed baseline noise (i.e., 22 for no regression and 30 for SCR) for one or both sessions, respectively. Pearson’s correlation coefficient, its confidence bounds, and p-values between days are displayed in the upper left corner of each scatter plot. Data points are labeled with the subject number, and the red dashed line indicates the confidence bounds.

On a single-subject level (see A), task-evoked brain activation became visible for nine out of 15 subjects, whereas six subjects showed only minimal or no activation (subjects 5, 6, 7, 8, 14, and 15). These qualitative results can be put into relation with the individual t-values (visible in Fig. 3 or 5), which were the lowest for the six subjects with weak activation. More specifically, the t-values for subjects with weak hemodynamic responses were in the range of 14 to 24 after SCR, which is below the threshold of 30 (as determined from baseline measurements). The lowest t-value of the other subjects was 31. Subsequently, the nine subjects with distinct spatial activation and t-values >30 are denoted “strong responders,” and the other six subjects with t-values ≤30 are “weak responders.”

### 3.2 Reproducibility

In the correlation plots in Fig. 3, the agreement between the test (session 1) and retest (session 2) sessions among all subjects is presented, taking into account t-values from single subjects. The test-retest agreement changed depending on the chromophore ($O_{2}\mathrm{Hb}$ or HHb, respectively) and signal processing step. The lowest agreement with correlations of 0.71/0.51 (left/right hand) was observed for [$O_{2}\mathrm{Hb}$] when no regression was applied and increased after SCR to 0.81/0.54. Correlation for [HHb] was only slightly higher for SCR over NR with 0.81/0.70 to 0.81/0.64. A distinct difference between right and left hand grasping was observed, with the left hand task always scoring more than 0.1 points less in correlation coefficients. The MAE% in Table 1 supports this observation, as the MAE% was generally lower for the right hand grasping task than the left hand grasping task (20.1% versus 13.4% for SCR $O_{2}\mathrm{Hb}$).

**Table 1:** ICC and MAE% single and average ICCs are given for ROIs above contralateral M1 for right (ROI 1) and left (ROI 5) hand grasping.^17^ Results for NR and SCR data are shown. The t-values of the runs per session were averaged. In brackets, the 95% confidence intervals are given. MAE% indicates the mean absolute scaled error relative to the range of the data.

		Right hand			Left hand		
		${\mathrm{ICC}}_{\text{single}}$	${\mathrm{ICC}}_{\text{average}}$	MAE% (%)	${\mathrm{ICC}}_{\text{single}}$	${\mathrm{ICC}}_{\text{average}}$	MAE% (%)
NR	$O_{2}\mathrm{Hb}$	0.64 [0.43, 0.82]	0.88 [0.75, 0.95]	15.4	0.58 [0.37, 0.77]	0.85 [0.70, 0.93]	18.3
	HHb	0.80 [0.67, 0.90]	0.94 [0.89, 0.97]	13.2	0.65 [0.46, 0.82]	0.88 [0.77, 0.95]	19.7
SCR	$O_{2}\mathrm{Hb}$	0.79 [0.65, 0.90]	0.94 [0.88, 0.97]	13.4	0.62 [0.42, 0.80]	0.87 [0.74, 0.94]	20.1
	HHb	0.81 [0.68, 0.91]	0.94 [0.89, 0.98]	13.3	0.73 [0.57, 0.87]	0.92 [0.84, 0.96]	16.8

Test-retest reliability based on ICC is presented in Table 1. The contralateral fNIRS channel above M1 was investigated for each task, i.e., left M1 for the right hand task and right M1 for the left hand task. The ICC values ranged between 0.58 and 0.81 for the single measurements and 0.85 and 0.94 for the averaged metric. It was found that the ICCs depend on the chromophore ([$O_{2}\mathrm{Hb}$] and [HHb]) and processing steps (NR and SCR). There was a trend that ICCs were higher after SCR in comparison with NR, as the ${\mathrm{ICC}}_{\text{single}}$ values were fair–good for NR and increased to fair–excellent for SCR. The ${\mathrm{ICC}}_{\text{average}}$ was excellent for all investigated combinations. The ICCs were higher for the right hand than the left hand grasping task.

### 3.3 Linear Mixed Effects Model

The influence of different variables on t-value estimation was determined by two linear-mixed effects models applied on [$O_{2}\mathrm{Hb}$] and [HHb].

Analysis of variance of [$O_{2}\mathrm{Hb}$] revealed a strong significant main effect of the hand*channel interaction ($F_{\left(7,827\right)}=21.78$, p<.001). Also, signal strength ($F_{\left(1,235\right)}=19.88$, p<.001), run ($F_{\left(3,843\right)}=3.04$, p<.05), and MW amplitude ($F_{\left(1,925\right)}=5.80$, p<.05) were found to have a significant effect on t-values. For the right hand grasping ($O_{2}\mathrm{Hb}$), the highest activation was found in left M1, and the lowest activity was detected in right M1. The left hand condition ($O_{2}\mathrm{Hb}$) had the highest activity in right M1 and the lowest in left M1.

The results for HHb were similar to $O_{2}\mathrm{Hb}$. A strong significant main effect of hand*channel interaction was found ($F_{\left(7,827\right)}=15.52$, p<.001). Signal strength ($F_{\left(1,239\right)}=13.47$, p<.001), run ($F_{\left(3,842\right)}=6.23$, p<.001) and MW amplitude ($F_{\left(1,921\right)}=17.68$, p<.001) had a highly significant effect on t-values. The highest activity for the right hand (HHb) was found in left M1, and the lowest activity was detected in right M1. For the left hand grasping (HHb), the highest activity was in right M1 and the lowest in left M1.

### 3.4 Confounding Factors

**Fig. 4:**
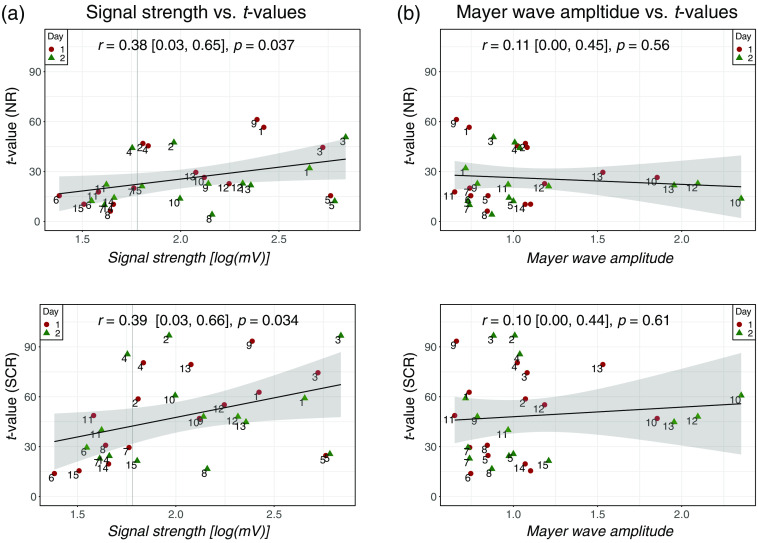
Influence of signal strength and MW amplitude on brain activity estimates. (a) t-values are plotted against signal magnitude for NR and SCR signals. (b) t-values are plotted against the median MW amplitude for NR and SCR signals. The highest t-values of the two runs were averaged per session. The labels correspond to the subject number. Pearson’s correlation coefficient, confidence bounds, and p-values are displayed in the upper left corner of each scatter plot.

The effect of signal strength and MW amplitude on t-values is visualized in Fig. 4. In Fig. 4(a), the raw signal magnitudes are plotted against t-values, with the maximal values for all runs averaged per subject. For better visibility, log-transformation was applied on the signal strength. A trend can be observed that smaller signal strength correlates with smaller t-values. In particular, four out of six weak responders (subjects 6, 7, 14, and 15) had low signal strength. An outlier was subject 5, which had low t-values but high signal strength. In Fig. 4(b), MW amplitude is compared with t-values. When ignoring the subjects with low signal strength (subjects 6, 7, 14, and 15), a negative correlation between t-values and MW amplitude is observed.

**Fig. 5:**
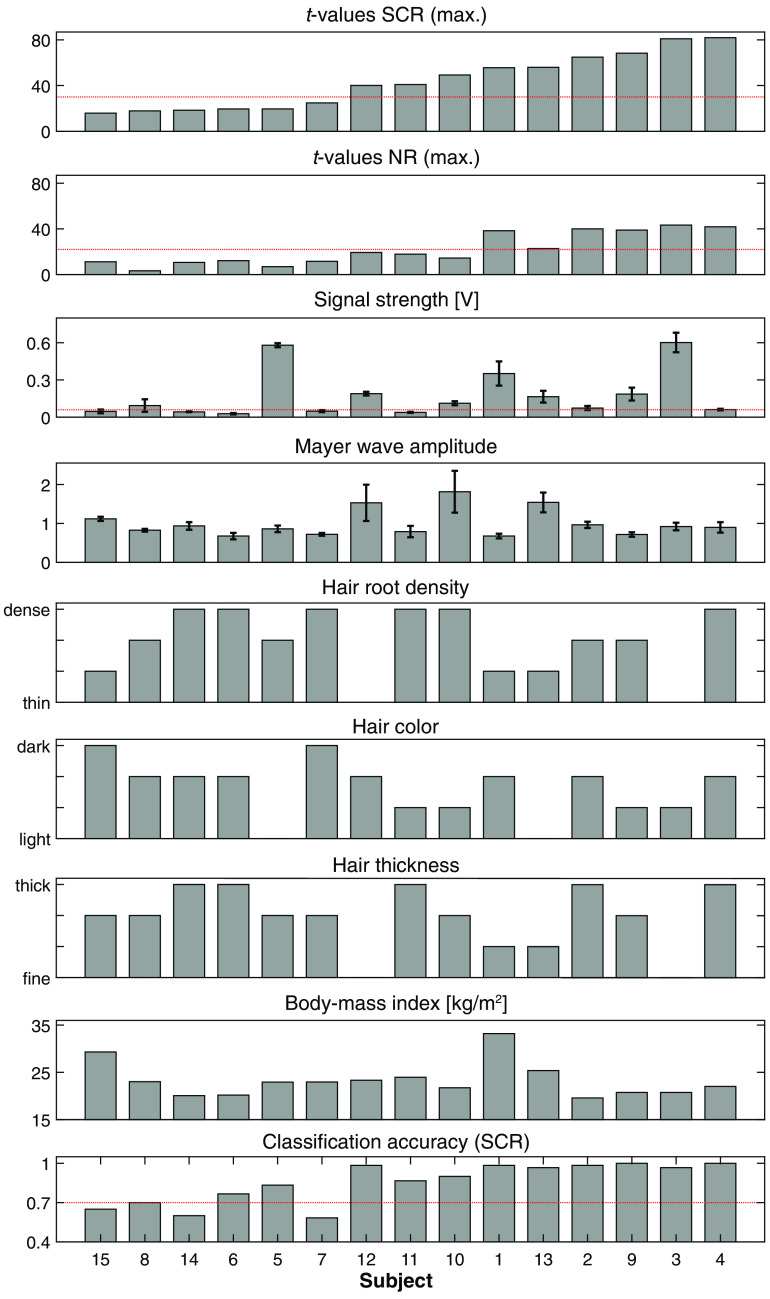
Subject-specific signal assessment. Data are sorted according to SCR t-values from lowest to highest subject-specific values (first row). The maximal t-value of the four runs per subject were averaged. The red lines for t-values indicate the threshold of 30 and 22 for the separation of subjects into strong and weak activators, which were found based on a 5% level of significance above chance. Signal strength is given in voltage, and the red line defines the threshold of 0.06 V, which is required for measurements with sufficient quality. In the last row, classification accuracy per subject averaged over the two testing runs is shown, with the red line at 70% indicating the 1% level of significance above chance. SCR, short-channel regression; NR, no regression.

Figure 5 further presents influencing factors in relation to t-values on a single-subject level. It again becomes visible that the subjects with high t-values often have high signal strength and low Mayer-wave amplitudes. High t-values are closely linked to high signal strength, which is dependent on hair characteristics: subjects with dense and dark hair tend to have lower signal strength than those with blonde and thin hair. A correlation between classification accuracy and t-values is observed, with subjects having high t-values also scoring high classification accuracy.

### 3.5 Classification

**Fig. 6:**
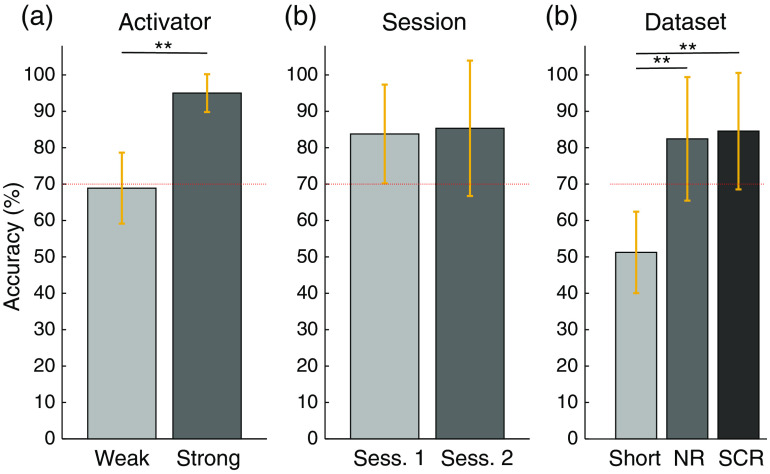
Classification accuracies. (a) Strong versus weak activators (SCR signals). (b) First versus second sessions (SCR signals). (c) Different input signals for the classifier. In particular, signals from SS and long-separation NR channels, as well as SCR signals, were used as input for the classifier pipeline. The red line indicates the 1% significance level for the classifier.^52^ **: p<0.001 using a two-tailed, paired t-test.

Classification accuracies were in good agreement with the results from the previous analyses: strong responders had high accuracies and weak responders had low accuracies. In the bottom line of Fig. 5, the single-subject classification accuracy graphically presents this trend as a dependency of t-values. There were seven subjects with accuracies >95% (subjects 1, 2, 3, 4, 9, 12, and 13). When separating the subjects into strong and weak responders, a significant difference between the two groups is observed [95% versus 69%, Fig. 6(a)]. No significant difference was observed between the classification accuracies of the test and retest sessions [Fig. 6(b)]. When feeding different input data to the classifier, there was no significant advantage of using SCR over NR data in terms of classification accuracy. When only SS data were used as input for the classifier, as a way to validate the absence of brain activity in the SS measurements, the accuracy was close to 50%, which corresponds to chance level and confirms the assumption that no brain activity is present in SS channels.

## 4 Discussion

In this work, we examined the reproducibility of fNIRS measurements during an active grasping task and the influence of SCR on it. We specifically investigated reproducibility on a single-subject level, which has not been presented yet in fNIRS. Furthermore, we linked reproducibility with BCI classification and factors affecting the estimation of brain activity.

On a group level, we successfully detected task-evoked cerebral activation and demonstrated the canonical hemispheric dominance, with the ROIs above the contralateral M1 (ROI 1 and 5) exhibiting the strongest hemodynamic responses for the right and left hand grasping tasks. In contrast, the more frontal (ROI 4 and 8) and ipsilateral (ROI 5+6+7+8 or 1+2+3+4) regions showed no or much reduced activation depending on the moved hand (left and right). These observed activity patterns are as expected and aligned with the fNIRS literature,^16^^,^^53^^–^^56^ in which similarly highest activation was observed over the contralateral M1, weaker activation over the ipsilateral M1, and weakest activation in frontal regions. Applying SCR was found to improve signal variability and to increase the spatial specificity of the hemodynamic response. Although more localized brain activation after reducing systemic activity has been reported,^16^^,^^55^^–^^57^ we demonstrated a high degree of reproducibility on a single-subject level for the first time. Hence, we provide crucial additional evidence for the efficacy of SCR. The change in t-values was more distinct for $O_{2}\mathrm{Hb}$ than for HHb, which is consistent with previous studies and a consequence of the stronger influence of systemic activity on $O_{2}\mathrm{Hb}$.^58^^,^^59^

We thoroughly characterized the reproducibility of fNIRS measurements after minimizing factors that could affect them. Specifically, we set up a simple hand grasping task to reduce physiological changes (e.g., blood pressure changes from the task execution) and obtained a well-localized and distinct brain activity pattern. We placed the fNIRS optodes over the M1-hotspots as identified by TMS, which reduced the influence of inconsistent optode placement.^60^ The used hardware was designed for measurements over the motor areas and the simultaneous capturing of physiological changes from very short source–detector separations (7.5 mm). Due to these technical features, our results indicate a high reproducibility at both the single-subject level and the group level. This is expressed not only by high correlation coefficients of t-values between the test and retest sessions but also by high ICCs. Although we showed that a high reproducibility in fNIRS signals can be achieved, it also becomes visible that other factors can lead to an unexplained variability in the brain activity estimates. More specifically, for the left hand grasping task, the ${\mathrm{ICC}}_{\text{single}}$ were distinctly smaller than for the right hand grasping task. The origin of these differences should be further investigated, but it is assumed to be a consequence of the dominance of right-handed subjects (i.e., 12 out of 15 subjects) leading to a more diffuse response in the left hemisphere.^61^^–^^63^ Similarly, the larger MAE% observed during the left-hand grasping task is expected to originate from the major part of the subjects being right-handed.

We adopted the concept of “strong” and “weak” responders from Saager et al.^64^ (denoted there as “good” and “poor” activators) and grouped the subjects according to their maximal t-values, with a threshold of t≥30 (after SCR) for the strong activators. This threshold was substantiated by the visual inspection of the spatial patterns of the hemodynamic responses and the statistically significant difference in classification accuracies when removing weak responders (69% versus 95%). Similarly, other works reported the inability to recover a hemodynamic response in some subjects during similar motor execution tasks. Yücel et al.^65^ anecdotally mentioned that they were not able to recover a hemodynamic response in 10% of the subjects. Franceschini et al.^66^ did not manage to detect a significant activation for 3/8, 6/10, and 8/11 subjects for visual stimulation, cognitive stimulation, and finger-tapping, respectively. Zimmermann et al.^67^ did not observe a significant activation in 1/7 subjects for an active grasping task. In electroencephalography (EEG), the notion of “BCI illiterates” or “nonresponders”^68^ is well known, also addressing the issue of persons for which insufficiently strong brain signals are captured. We suggest considering the concept of strong and weak responders in future fNIRS studies and reporting (maximal) t-values as a marker for the inherent presence of brain activation.

As the main driver for the separation into strong or weak responders, we observed two critical factors. First, five of the six weak responders had a low raw signal intensity below 0.06 V, which corresponds to an SNR below 40 dB and which is often suggested as a threshold for reliable fNIRS measurements.^23^^,^^33^^,^^69^ Thus, it can be claimed that high optical sensitivity is a crucial premise for detecting brain activation reliably. This is an essential finding for fNIRS instrumentation in general, which implies that the optical signal strength should be determined at the beginning of each measurement. We suggest using the optical signal strength as an exclusion criterion when performing applied research (e.g., robot control by a BCI and clinical studies) and reporting the level of raw signal strength in future publications. Performing screening prior to a neuroscientific study has been proposed,^70^^–^^72^ and metrics related to signal quality, such as the scalp coupling index,^73^ the light-tissue coupling index,^74^ or the signal quality index,^75^ could be adapted. Second, MWs showed a significant effect on t-values, with all subjects with low MW amplitudes exhibiting high t-values, thereby confirming the relevant literature.^76^^,^^77^ We showed that the t-values increased strongly when SCR was applied. The applied SCR method (${\mathrm{nnGLM}}^{\mathrm{multiSS}}$) specifically considered MW oscillations^27^ and therefore reduced the effect of MW oscillations on the detection of brain activity.

For the future use of fNIRS for (out-of-the-lab) BCI applications, we trained a classifier on the first run and tested it on the unseen second run for each session. A relatively high classification accuracy of 85% was obtained over all subjects and sessions, which is in a similar range as other fNIRS studies investigating motor execution.^12^^,^^50^^,^^78^^–^^82^ Three out of the 15 subjects (20% of the subjects) did not exceed the 70% significance threshold on a binary classification task.^52^ When separating subjects into strong and weak responders, the former group achieved an average accuracy of 95%. Because the t-values and the classifier accuracies were directly related in our dataset, it should be considered to determine a suitable threshold with a 5% significance level above chance and implement screening sessions at the beginning of fNIRS studies to detect subjects with low t-values. These subjects are expected to be unsuitable for neuroscience studies or BCI applications. It was surprising to observe no significant difference in classification accuracy between not-regressed or SCR input signals for the classifier. This finding indicates that, for robust BCI settings, the additional use of SS channels may not be of fundamental importance. Although this finding requires further research, it must be remembered that, for applications that assess origin, patterns, or magnitudes of brain activity, the inclusion of SS channels is essential.^27^

The TMS localization was performed to investigate the reproducibility of measurements and ensure that the M1-hotspot was precisely determined for each subject. Although performing TMS localization in real-world applications would be impractical, it was important in the context of this study to maximize signal response and quality. As possible alternatives to TMS, approaches such as unguided optode placement should be considered. Because the hair characteristics (density and color) had a strong influence on the ability to extract strong brain activation in the statistical analysis, it is especially important to make sure that the fNIRS instrument optimally copes with hair^12^^,^^83^—in addition to a careful experimental design.^84^^,^^85^ A simple and optimal optode placement with minimal hair obstructing the light propagation is crucial to achieving a high SNR.^84^ This is a general challenge of wearable fNIRS systems due to larger diameters of the lightguides compared with laser-based systems and the modular optode structure to facilitate multidistance measurements. One strength of optoHIVE is that it uses highly sensitive detectors, which promises to better cope with hair.

In this study, we did not obtain spatial-dependent values of scalp thickness, bone density, or skull thickness, which could influence the $O_{2}\mathrm{Hb}$ and HHb estimates or the statistical analysis. We expect a correlation between these parameters and t-value in the statistical analysis as has been shown in the relevant literature,^29^^,^^86^^,^^87^ giving a possible explanation for residuals in the GLM. However, in terms of detecting an activation, which is the target of a BCI, we consider that variations of these parameters lead only to a moderate deviation from the correct differential pathlength factor and have a negligible influence on the results. The simplicity of the hand grasping task could have introduced occasional delays or variations in the hemodynamic responses due to momentary inattention of the subject. Furthermore, classification was performed only sessionwise and based on the entire trial of 16 s. The latter is practicable for pseudo-online classification in the frame of this work, but for a real-time BCI, the decision window should be reduced to a few seconds after the task onset.^88^ As an ultimate goal, transfer learning from one session to another,^89^^,^^90^ as well as asynchronous BCI settings (i.e., the task onset is not known),^91^ should be addressed to make the step toward in-home applications.

## 5 Conclusion

In this work, we demonstrated that fNIRS measurements are reproducible on a single-subject level when fulfilling certain prerequisites (i.e., localization of M1 with TMS, careful selection of study protocol, and optimized hardware). We separated the measured subjects into strong and weak responders based on the quality of the hemodynamic response and showed that higher test-retest reliability and classification accuracy are obtained for the strong responders without and more distinctly with SCR applied. Raw optical signal strength and MWs were found to be the major determinants of reproducibility. Therefore, to ensure robustness in fNIRS applications and that sufficient brain activity is captured, we suggest screening each subject once with regard to raw optical signal strength and t-values prior to the first experiment. Based on optoHIVE, a wearable and highly sensitive fNIRS instrument with the integrated ability to perform effective SCR, this work opens a new dimension of fNIRS, i.e., its reliable application in single subjects in everyday environment and consequently in the clinical and BCI fields.

### 6 Appendices

### 6.1 A Appendix

**Fig. 7:**
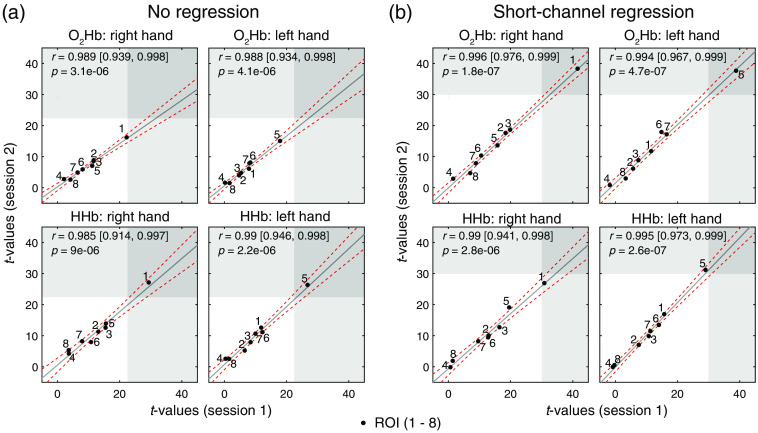
Group-level correlation of the eight ROIs between test and retest sessions. Reproducibility of t-values between the two sessions was assessed for [$O_{2}\mathrm{Hb}$] and [HHb] of the right and left hand grasping tasks. t-values give an indication of the quality of the measured hemodynamic response. For each task, the correlation plots for the eight ROIs averaged over all subjects are shown. (a) Results for NR t-values. (b) Results for regressed (after SCR). Gray areas indicate the t-value range of statistical significance (i.e., 22 for no regression and 30 for SCR). Pearson’s correlation coefficient, its confidence bounds, and p-values between days are displayed in the upper left corner of each scatter plot. Data points are labeled with the ROI number, and the red dashed line indicates the confidence bounds.

In Fig. 7, correlation between the test and retest sessions is shown on the group level with the average t-values over all subjects per ROI. This plot confirms the findings from Plichta et al.^60^ that fNIRS are highly reproducible across sessions for group-averaged optode locations.

**Fig. 8:**
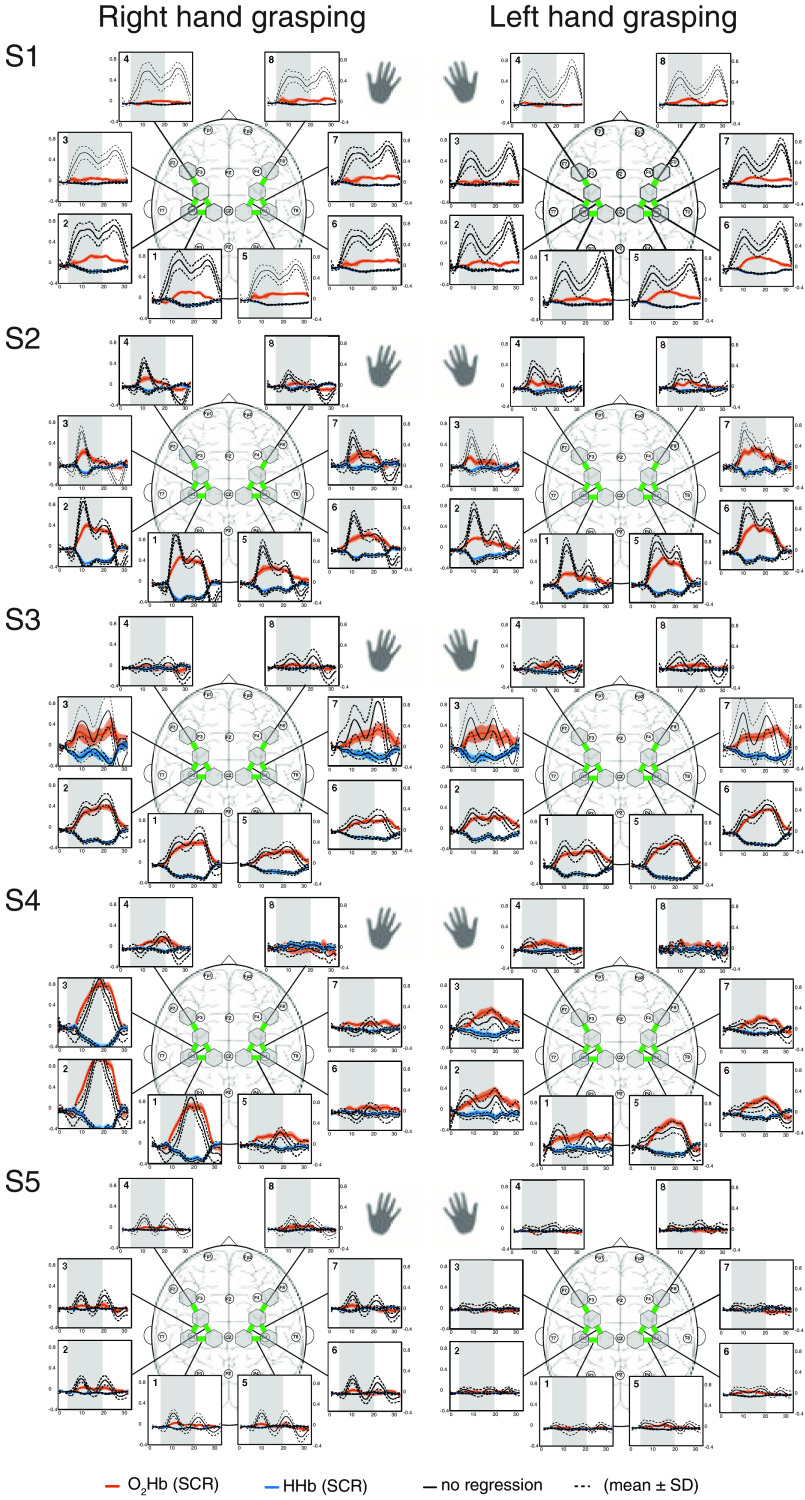
Block average of [$O_{2}\mathrm{Hb}$] and [HHb] for subjects 1 to 5. The hemodynamic responses of the four runs per subject were averaged (mean±SD) for each ROI. The spatial patterns for the right and left hand grasping tasks are shown. The gray bars indicate the task period when grasping with either the left or right hands was conducted. Units are in μM.

**Fig. 9:**
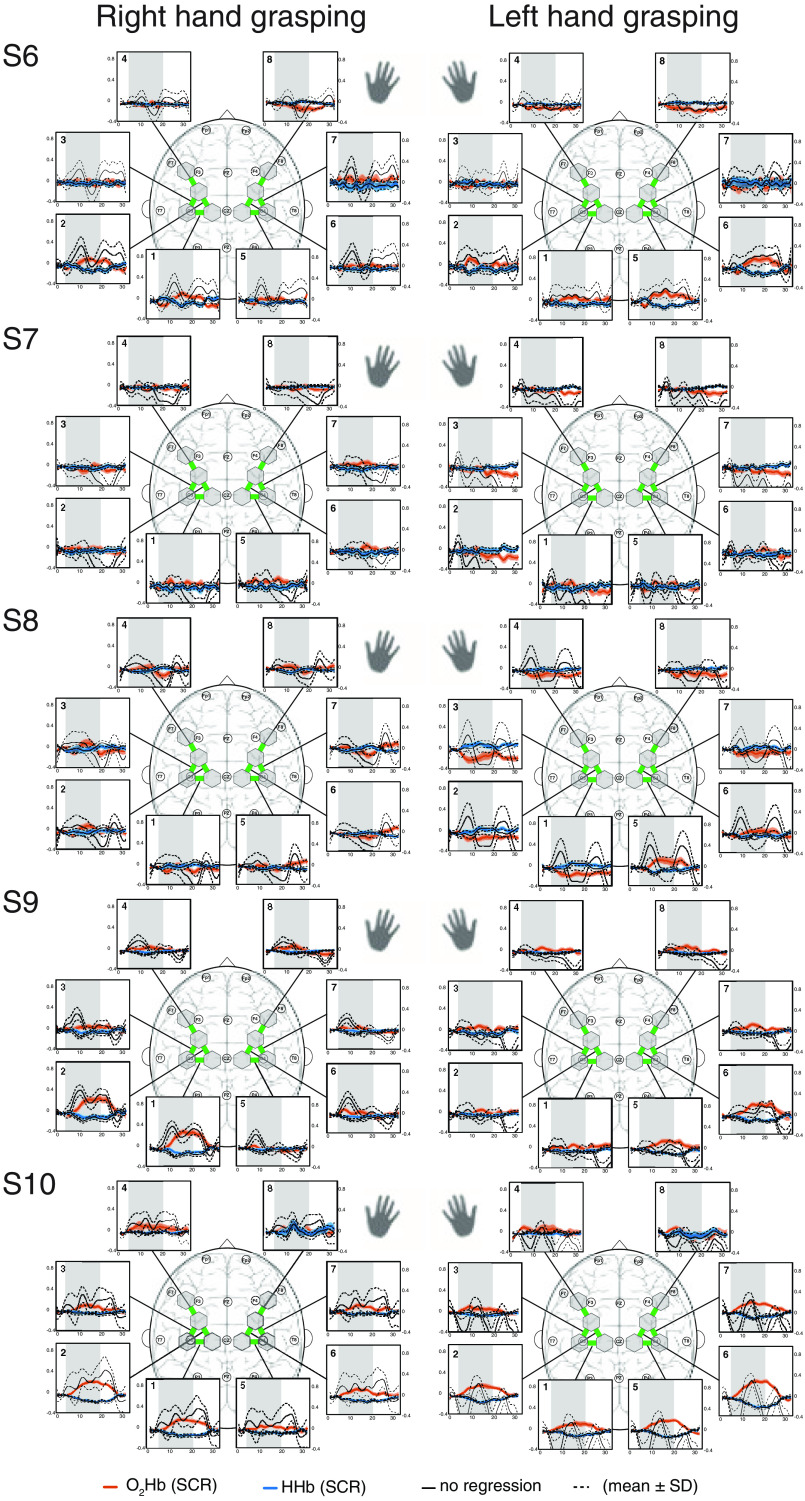
Block average of [$O_{2}\mathrm{Hb}$] and [HHb] for subjects 6 to 10. The hemodynamic responses of the four runs per subject were averaged (mean±SD) for each ROI. The spatial patterns for the right and left hand grasping tasks are shown. The gray bars indicate the task period when grasping with either the left or the right hand was conducted. Units are in μM.

**Fig. 10:**
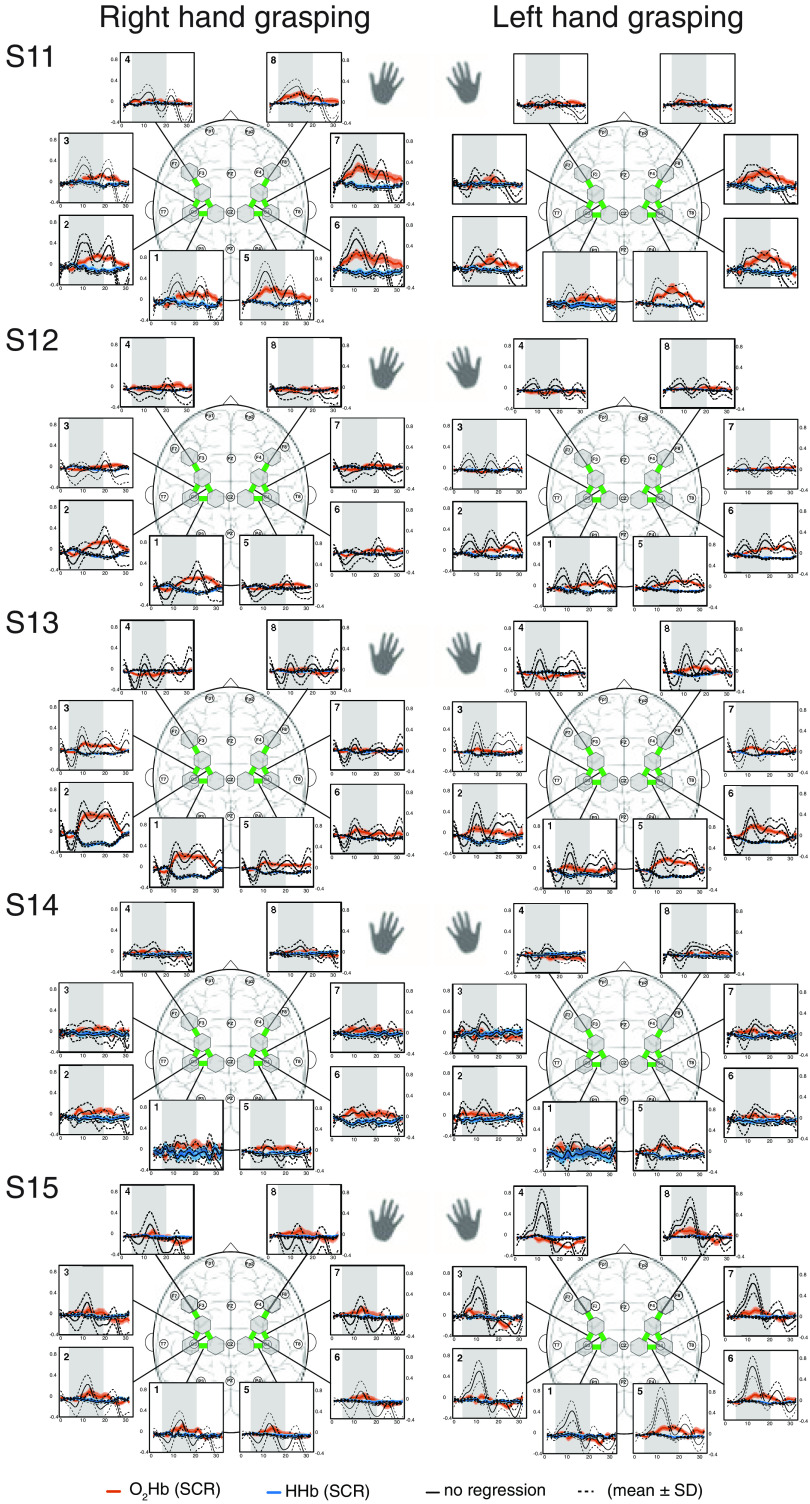
Block average of [$O_{2}\mathrm{Hb}$] and [HHb] for subjects 11 to 15. The hemodynamic responses of the four runs per subject were averaged (mean±SD) for each ROI. The spatial patterns for the right and left hand grasping tasks are shown. The gray bars indicate the task period when grasping with either the left or the right hand was conducted. Units are in μM.

In Figs. 8–10, the block averages of each subjects are shown for the right and left hand grasping tasks. Individual patterns for individual subjects become apparent. For example, there were subjects (e.g., S2, S3, and S4) exhibiting strong and easily visible hemodynamic responses, others (e.g., S1, S5, S15) having strong task-evoked systemic activity, and some (e.g., S7, S8, S14) showing no to minimal activation.

## Data Availability

Data and code from this manuscript are available from the authors upon reasonable request and after filling out a formal data sharing agreement.
